# Cryo-EM structure of the monomeric *Rhodobacter sphaeroides* RC–LH1 core complex at 2.5 Å

**DOI:** 10.1042/BCJ20210631

**Published:** 2021-10-21

**Authors:** Pu Qian, David J.K. Swainsbury, Tristan I. Croll, Jack H. Salisbury, Elizabeth C. Martin, Philip J. Jackson, Andrew Hitchcock, Pablo Castro-Hartmann, Kasim Sader, C. Neil Hunter

**Affiliations:** 1 Materials and Structural Analysis, Thermo Fisher Scientific, Achtseweg Noord 5, 5651 GG Eindhoven, Netherlands; 2 Department of Molecular Biology and Biotechnology, University of Sheffield, Sheffield, U.K.; 3 Cambridge Institute for Medical Research, University of Cambridge, Cambridge CB2 0XY, U.K.; 4 Department of Chemical and Biological Engineering, University of Sheffield, Sheffield, U.K.

**Keywords:** bacteriochlorophyll, carotenoid, light harvesting, photosynthesis, quinone, reaction centre

## Abstract

Reaction centre light-harvesting 1 (RC–LH1) complexes are the essential components of bacterial photosynthesis. The membrane-intrinsic LH1 complex absorbs light and the energy migrates to an enclosed RC where a succession of electron and proton transfers conserves the energy as a quinol, which is exported to the cytochrome *bc*_1_ complex. In some RC–LH1 variants quinols can diffuse through small pores in a fully circular, 16-subunit LH1 ring, while in others missing LH1 subunits create a gap for quinol export. We used cryogenic electron microscopy to obtain a 2.5 Å resolution structure of one such RC–LH1, a monomeric complex from *Rhodobacter sphaeroides.* The structure shows that the RC is partly enclosed by a 14-subunit LH1 ring in which each αβ heterodimer binds two bacteriochlorophylls and, unusually for currently reported complexes, two carotenoids rather than one. Although the extra carotenoids confer an advantage in terms of photoprotection and light harvesting, they could impede passage of quinones through small, transient pores in the LH1 ring, necessitating a mechanism to create a dedicated quinone channel. The structure shows that two transmembrane proteins play a part in stabilising an open ring structure; one of these components, the PufX polypeptide, is augmented by a hitherto undescribed protein subunit we designate as protein-Y, which lies against the transmembrane regions of the thirteenth and fourteenth LH1α polypeptides. Protein-Y prevents LH1 subunits 11–14 adjacent to the RC Q_B_ site from bending inwards towards the RC and, with PufX preventing complete encirclement of the RC, this pair of polypeptides ensures unhindered quinone diffusion.

## Introduction

The reaction centre light-harvesting complex 1 (RC–LH1) core complex is essential for bacterial photosynthesis. The energy of light absorbed by the LH1 antenna migrates to the RC, where a charge separation is followed by electron, then proton transfers to form a doubly reduced and protonated acceptor, ubiquinol. This molecule must leave the RC, traversing the surrounding LH1 ring and then diffusing a short distance to the cytochrome *bc*_1_ complex where ubiquinol is oxidised, creating a proton motive force that drives ATP synthesis [1–3]. Several structures of these RC-LH1 membrane protein complexes from various phototrophic bacteria show that the RC is surrounded by a circular LH1 antenna complex, yet the RC can sustain cyclic electron flow and photosynthetic growth. Thus, there must be small pores in the LH1 structure that permit quinones and quinols to cross the LH1 complex, and indeed recent structures of such RC–LH1 complexes, where the RC is surrounded by 16 LH1 αβ subunits, show that such pores exist [4–7]. Another structure, from the phototropic bacterium *Rhodobacter (Rba.) veldkampii*, shows how the introduction of a transmembrane PufX polypeptide into the LH1 complex creates a large opening in the LH1 ring that would permit quinones and quinols to enter and leave the complex [8]. In the related bacterium *Rba.* *sphaeroides*, PufX-minus mutants cannot grow photosynthetically because they have a completely closed ring that impedes quinone traffic [9], but there is no structure of the RC–LH1–PufX complex to show how the quinone channel is formed. Yet, there is a greater imperative to form a dedicated channel for quinones in the case of *Rba. sphaeroides* because, unlike *Rba. veldkampii,* its LH1 has nearly twice the carotenoid content [10], which was proposed to occlude any small pores within the LH1 ring [11]. We have suggested that while the increased carotenoid content could come at some cost in terms of hindering quinone traffic, any disadvantages would be more than offset by extra carotenoids harvesting more visible light and offering enhanced photoprotection [11]. These carotenoids would also augment light-harvesting capacity, which would likely increase turnover at the RC Q_B_ site and necessitate more efficient quinone diffusion to and from the RC.

Previously we used X-ray crystallography to obtain a medium-resolution structure of the RC–LH1–PufX complex in its dimeric form [12]. The 8 Å resolution of the electron density map allowed fits of LH1 subunits round the RC, and the structure revealed a gap in the 14-subunit LH1 ring; unassigned electron density was attributed to the PufX polypeptide. In the intervening time, cryogenic electron microscopy (cryo-EM) has transformed our ability to obtain high-resolution structures of protein complexes, and we applied this method to determine the structure of the monomeric RC–LH1–PufX complex. Our structure shows how PufX prevents full encirclement of the RC and it reveals a hitherto undescribed polypeptide, designated as protein-Y, which binds to the RC-L subunit and to the inside face of the LH1 complex near the RC Q_B_ site. Protein-Y forms a stable internal channel for quinones to move between the inner face of protein-Y and the RC and, with PufX, provides a route between the exterior of the complex and the RC. This extra protein-Y component could represent a beneficial adaption that allows enhanced light harvesting and photoprotection from the increased carotenoid complement, coupled to an optimised protein-Y/PufX-mediated strategy for rapid quinone diffusion.

## Materials and methods

### Cell culture

The PufX R53L mutant of *Rba. sphaeroides* was used to abolish the formation of RC–LH1 dimers, thus ensuring a homogeneous population of monomeric complexes [9]. This strain was cultured photosynthetically in M22+ medium under illumination of 150 µmol of photons m^−2^ s^−1^ using Osram 116 W halogen bulbs at 30°C in 1.4 L cell culture flask. Cells were harvested by centrifugation at 3 290×***g*** for 30 min after the culture reached an optical density (OD) at 680 nm of 1.6. The harvested cells were stored at −80°C before use.

### Protein purification

Cells were washed with working buffer (20 mM HEPES, pH 7.8), mixed with a few grains of DNaseI and a few crystals of MgCl_2_, then passed through a chilled French Press three times under a pressure of 18 000 psi. The suspension of broken cells was layered onto a two-step sucrose density gradient (15/40% (w/w) sucrose) then centrifuged for 5 h at 100 000×***g***. Photosynthetic membranes sitting at the 15–40% sucrose interface, were collected, then diluted three times using working buffer and pelleted by centrifugation for 1 h at 235 000×***g*** (45 000 rpm, Beckman 45Ti rotor). The pelleted membrane was resuspended in working buffer to an absorbance of 100 at 874 nm. The final mixture for protein solubilisation was adjusted to 10 ml, with an absorbance of 60 at 874 nm and 3% β-DDM (w/w), and stirred in dark for 30 min at 4°C. Centrifugation for 1 h at 211 000×***g*** removed unsolubilised material and the clear supernatant was loaded on to a five-step sucrose density gradient (20/21.25/22.5/23.75/25% (w/w) sucrose in running buffer, which is working buffer containing 0.03% w/w β-DDM), and spun for 16 h at 125 000×***g*** (27 000 rpm, Beckman SW41 rotor). The band containing monomeric core complexes was collected and applied to an ion-exchange column (DEAE-Sepharose, Sigma) pre-equilibrated in running buffer. The column was washed using 2 column volumes of running buffer followed by stepwise washing to 120 mM NaCl. A 100 ml gradient from 120 to 300 mM NaCl was used to elute protein from the column. Core complex fractions, which eluted at 250 mM NaCl, were collected and concentrated to 1 ml then loaded onto a Superdex 200 gel filtration column (GE Healthcare). Eluted fractions were monitored by the A_874_/A_280_ absorbance ratio, and those with a ratio higher than 1.7 were pooled and concentrated for cryo-EM grid preparation.

### Cryo-EM data collection

The protein concentration was adjusted to an absorbance of 10 at 874 nm. 3.0 μl protein solution was applied to a home-made graphene oxide (Sigma–Aldrich, U.S.A.) grid (Quantifoil grid R1.2/1.3, 300 mesh Cu). Cryo-EM grids were prepared using a FEI MK4 Vitrobot. Parameters were set as follows: wait time 30 s, blotting time 2.5 s, blot force 3, sample chamber humidity 99%, sample chamber temperature 4°C. The grid was plunged into liquid ethane cooled by liquid nitrogen and stored in liquid nitrogen before use.

Data were collected at the Cambridge Pharmaceutical CryoEM Consortium on a Thermofisher Scientific Titan Krios G3i Cryo-EM equipped with a Falcon 4 direct electron detector [13]. The microscope was operated at 300 kV accelerating voltage, at a nominal magnification of 120 k, corresponding to a pixel size of 0.65 Å at the specimen level. The detector was operated in counting mode. A total dose of 44.99 electrons per Å^2^ was fractionated to 42 frames within 12.21 s exposure time, resulting in an electron dose of 1.07 e^−^/Å^2^/frame. In total, 4 859 movies were collected with defocus values varied from 0.8 to 2.2 µm. A typical cryo-EM image after motion correction is shown in Supplementary Figure S1A.

### Data processing

RELION 3.1 [14] was used for image processing. Beam-induced movements of specimen recorded on individual frames were corrected using RELION's built-in motioncorr2 with 5 × 5 patches. CTF parameters were determined using CTFFIND4.1 [15]. Particle coordinates for the motion corrected images were determined using cisTEM [16]. The coordinates were transferred to RELION for particle picking. In total, 1 057 624 particles were picked with a box size of 380 × 380, corresponding to a 24.7 nm square. These particles were subjected to reference-free two-dimensional classification. 651 879 (58.95%) particles from good 2D classes were selected for 3D classification. A monomeric RC–LH1–PufX model of *Rba. sphaeroides*, taken from the dimer structure obtained from X-ray crystallography (PDB 4V9G), was converted to a 3D map using Chimera [17], and was used as an initial model for maximum-likelihood-based 3D classification with a 60 Å low-pass filter applied. One best 3D class out of four, containing 205 613 particles (19.44%), was selected for high-resolution 3D reconstruction and refinement, resulting in a 2.9 Å resolution 3D map. After CTF refinement, including anisotropic magnification, beam-tilt, trefoil, 4th order aberration, per particle defocus and per-image astigmatism estimation, Bayesian polishing, performed with the default parameters provided by RELION, improved the resolution of the 3D map to 2.6 Å. The selected particles for the 3D refinement were re-extracted using a 512 × 512 box size for a final CTF refinement and Bayesian polishing, producing a 2.5 Å resolution map for modelling.

### Modelling and refinement

Initially a RC–LH1–PufX monomer, taken from the crystal structure of the dimeric RC–LH1–PufX from *Rba. sphaeroides* (PDB 4V9G), was fit into the cryo-EM map as a rigid body using the *fit in map* function of Chimera. Polypeptides and cofactors were then manually adjusted and real space refined in COOT [18]. At this stage all-*trans* carotenoid molecules were fitted into the LH1 ring based on the density map using Chimera, then real space refined in COOT. We defined a previously unassigned U-shaped transmembrane protein sitting between LH1 and the RC as protein-Y. For this newly identified protein, we first built a poly-alanine trace using COOT, then assigned a tentative *de novo* sequence to the best-resolved regions by adding side-chains in ISOLDE with consideration of both fit to density and physical environment. This tentative sequence was then used as the basis for a BLASTp search of the *Rba. sphaeroides* genome, which yielded a single clear candidate sequence (Rsp_7571; https://www.uniprot.org/uniprot/U5NME9). Modelling of the full sequence into the map yielded good agreement with the density, and the presence of these chains in the complex was confirmed by mass spectrometry (see below). After real-space refinement in ISOLDE [19], the final model was subjected to global refinement and minimisation using PHENIX [20]. The final refinement statistics are summarised in Supplementary Table S1. The quality of fit for the structural model within the electron density map was validated using EMRinger [21].

### Identification of Rsp_7571 by mass spectrometry

RC–LH1 complex (50 µg) purified from wild type *Rba. sphaeroides* was solubilised in 100 µl 2% (w/v) sodium dodecylsulfate, 40 mM Tris base, 60 mM DTT at 60°C for 5 min. Proteins were extracted by precipitation using a 2-D clean up kit (Cytiva) according to the manufacturer's protocol. After centrifugation (15 700×***g*** for 10 min) the protein pellet was dissolved in 50 µl 0.2% (v/v) formic acid containing 2 µg pepsin (Promega) and incubated at 37°C for 16 h. Pepsin was more effective than trypsin because there are multiple potential cleavage sites in Rsp_7571, whereas there is only a single cleavage site (R10) for trypsin in a position that would produce only one proteotypic fragment (Supplementary Figure S2). Following the addition of 2.5 µl 10% (v/v) trifluoroacetic acid, the digest was desalted using a C_18_ spin column (Thermo Scientific) and dried by vacuum centrifugation.

Peptides were analysed by nano-flow liquid chromatography coupled to a Q Exactive HF quadrupole-Orbitrap (Thermo Scientific) mass spectrometer as previously described [22] except that a 75 min gradient was used for peptide separation. Protein identification was performed by database searching using Byonic (v2.9.38, Protein Metrics) operating with the default parameters except that methionine sulfoxide (+15.19949 Da) was specified as a common variable modification (with a maximum of two per peptide). Cleavage sites were specified as N- and C-terminal to F, Y, W and L (semi-specific). The database was the *Rba. sphaeroides* reference proteome (www.uniprot.org/proteomes/UP000002703) downloaded on 28th April 2021.

## Results

### Identification of protein-Y by mass spectrometry

As part of a routine characterisation we used mass spectrometry to analyse a pepsin digest of RC–LH1 complexes purified from the wild-type strain. RC-H, RC-M, RC-L and PufX were all detected, as were the LH1-α and LH1-β subunits (not shown). In addition to these expected RC, LH1 and PufX polypeptides we found a new component, Rsp_7571, which we designated as protein-Y. The primary sequence of protein-Y (Supplementary Figure S2) indicates a transmembrane protein of 53 residues, with a molecular mass of 5 554 Da, predicted to consist of two helices connected by a short loop. We found identical homologues in three other strains of *Rba. sphaeroides* and two species of *Luteovulum* (another member of the Rhodobacteraceae). In keeping with the nomenclature for the RC-H subunit encoded outside of the *puf* operon, we propose to name the gene encoding protein-Y as *puyA*.

### Overall structure of the monomeric RC–LH1 complex

**Figure 1.**
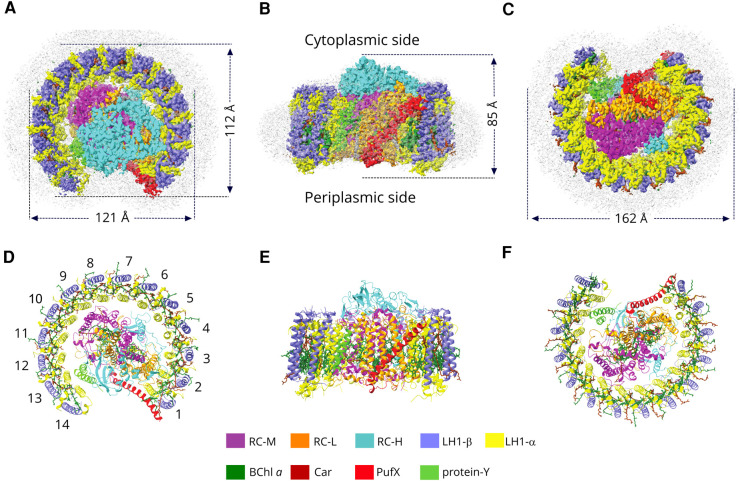
Cryo-EM structure of the RC–LH1 core complex monomer from *Rba. sphaeroides*. (**A**–**C**) Views of the RC–LH1 monomer density map, coloured as in the key at the bottom of the figure. Detergent and other disordered molecules are in grey. (**A**) View of the cytoplasmic face of the complex, showing the diameters of the long and short axes. (**B**) View in the plane of the membrane showing the height of the complex and the wide opening in the LH1 ring created by PufX and protein-Y. (**C**) Perpendicular view from the periplasmic side. (**D**–**F**) Ribbon models corresponding to (**A**–**C**), made using ChimeraX [17]; the LH1 subunits are numbered in (**D**).

The 2.5 Å structure of the monomeric RC–LH1 complex was determined by cryo-EM analysis (see Materials and Methods). Here we used the previously characterised PufX R53L mutation [9] to prevent the dimerisation of RC–LH1, ensuring a good yield of homogenous monomeric complexes for purification and structural analysis. Supplementary Figure S1 shows a typical cryo-EM image of the monomeric RC–LH1–PufX complex; selected 2D classes and the Fourier shell correlations show that the global resolution of the map is 2.5 Å. Supplementary Table S1 displays the information on data acquisition, model refinement and validation statistics, and fits of structural models of polypeptides and pigments of the monomeric RC–LH1–PufX complex within their respective cryo-EM densities are shown in Supplementary Figure S3. The colour-coded density map for the full complex is shown in Figure 1A–C, viewed in the plane of the membrane and from the cytoplasmic and periplasmic sides, along with the corresponding structural models of the complex (Figure 1D–F). The RC occupies the centre of the complex, and acts as the focal point for energy arriving from the surrounding LH1 antenna. There are many structures of the *Rba. sphaeroides* RC, all obtained from X-ray crystallography, so we took the opportunity to compare the present cryo-EM structure of the RC with a crystallographic structure (PDB: 3I4D) (Supplementary Figure S4A). Deviation in residue-residue distances between the two RC structures is only 0.44 Å on average, with small deviations between RC-L helices A and B and at the termini of RC-M and RC-H. These small differences can be attributed to interactions with the surrounding LH1 complex, which are absent from the crystal structure. The tails of the bacteriochlorophyll (BChl) and quinone cofactors vary in position but macrocycles/heads are well aligned (Supplementary Figure S4B).

Some phototrophic bacteria assemble a core complex in which the RC is fully encircled by a 16-subunit LH1 ring [4–7], but Figure 1 draws attention to the very open LH1 assembly found for the *Rba. sphaeroides* RC–LH1 monomer complex. There is a new component, protein-Y, clearly defined by its density in Figure 1A–C, which was not identified in the 8.5 Å resolution cryo-EM projection map of this complex [23], nor in the subsequent 8 Å resolution electron density map obtained from X-ray crystallography [12]. Here, protein-Y is inserted between LH1 αβ subunits 13 and 14 and the RC, with its transmembrane helices lying against the inner faces of the LH1α polypeptides, while PufX interrupts the LH1 ring and prevents the incorporation of more LH1 αβ subunits, which would cause LH1 to completely surround the RC.

### Protein–protein interactions that stabilise the LH1 antenna and promote its attachment to the RC

**Figure 2.**
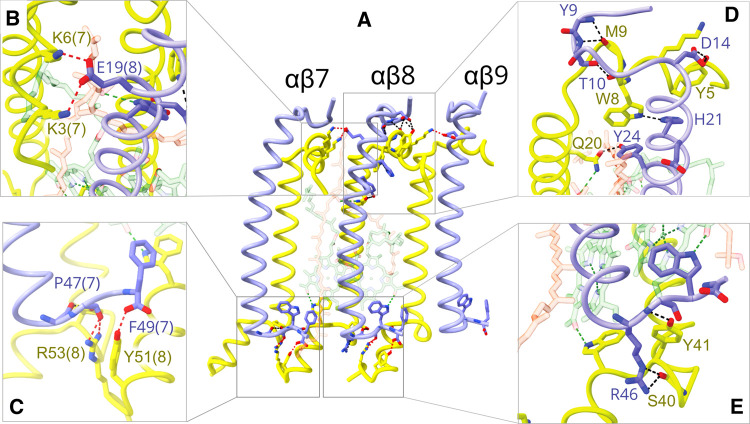
Inter- and intra-subunit interactions in the LH1 antenna of the monomeric RC–LH1–PufX complex. (**A**) LH1 αβ subunits 7–9, each with one α-polypeptide (yellow) and one β-polypeptide (cornflower blue); for clarity only αβ8 is shown with pigments, which are faded to emphasise the polypeptides. Inter- and intra-subunit hydrogen bonds are in red and black, respectively. Only residues involved in hydrogen bonds are labelled. Some bonds to BChls and carotenoids are also shown (green). Boxes **B**–**E** are expanded and reoriented views of the corresponding boxes in A. (**B**) Inter-subunit bonds near the cytoplasmic face of the complex. The numbers in parentheses indicate the subunit. (**C**) Inter-subunit bonds near the periplasmic face of the complex; the Trp near Phe49 was omitted for clarity. The hydrogen bond to Phe48 is to a main chain oxygen. (**D**) Intra-subunit bonds near the cytoplasmic face of the complex. (**E**) Intra-subunit bonds near the periplasmic face of the complex.

**Figure 3.**
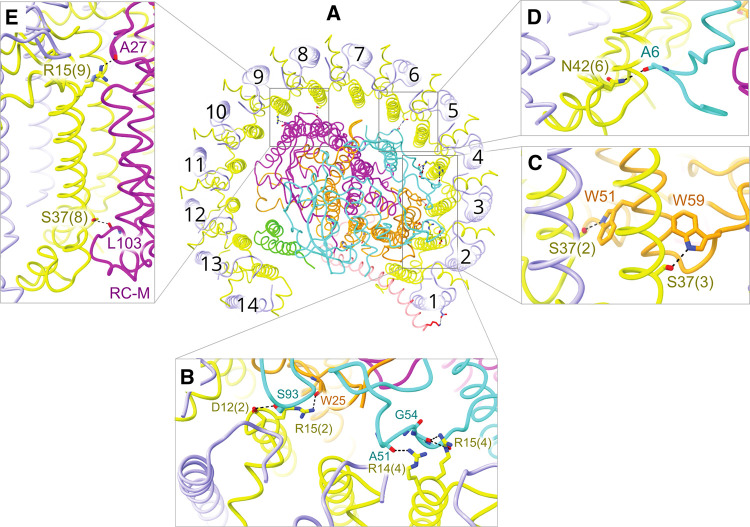
Interactions between the LH1 antenna and the RC. (**A**) The monomeric RC–LH1–PufX complex viewed from the cytoplasmic side. The 14 LH1 αβ subunits are numbered; BChls and carotenoids have been omitted for clarity, and the colours are as for Figure 3. RC-M is in magenta, RC-L is in orange and RC-H is in cyan. Boxes **B**–**E** show those parts of the LH1 ring that form hydrogen bonds to the exterior face of the RC. Only residues involved in hydrogen bonds are labelled.

**Figure 4.**
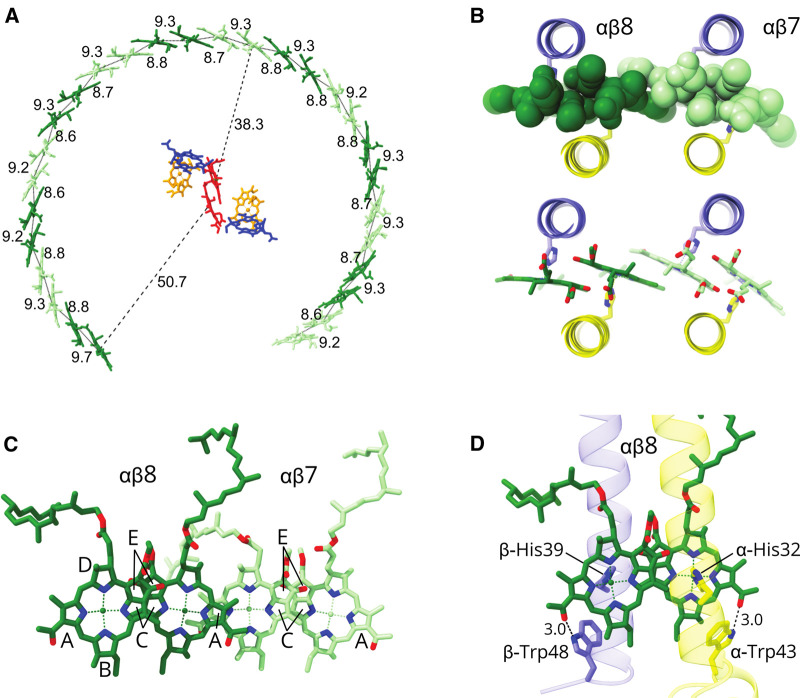
The bacteriochlorophylls and RC cofactors. Proteins and carotenoids are omitted for clarity. (**A**) View from the periplasmic side of the membrane showing the incomplete ring of 28 BChls, coloured in two shades of green to distinguish between pairs belonging to individual LH1 αβ subunits. Mg–Mg distances in Ångstroms for intra- and inter-subunit BChls are shown, and also for the nearest and furthest approach of BChls to the RC special pair of BChls (red). RC accessory BChls are in orange and bacteriopheophytins are in blue. (**B**) Two adjacent pairs of LH1 BChls viewed from the periplasmic side with the BChls in spacefill representation (upper image) to emphasise the close contacts between these pigments, and as stick models (lower image) to show the coordination by histidine residues from the LH1 α (yellow) and β (blue) polypeptides. (**C**) View in the plane of the membrane of two adjacent BChl pairs, coloured as in (**A**). Overlaps between the rings of the BChl macrocycle are shown. (**D**) A single LH1 subunit (αβ8) showing coordination by histidine residues and hydrogen bonds from C-terminal Trp residues to C3 acetyl carbonyls of the BChls.

Figure 2A shows three LH1 αβ subunits to illustrate the inter- and intra-subunit interactions that help to stabilise the LH1 ring. The transmembrane sections of LH1α and LH1β are flanked by N-and C-terminal domains that lie close to the cytoplasmic and periplasmic sides of the membrane, respectively. Figure 2B–E separate the various protein–protein interactions according to the side of the membrane and whether they are within or between subunits, showing that multiple bonds at the N- and C-terminal domains help to bind LH1α and β together, and contribute to the formation of an oligomeric LH1 assembly. In addition, the BChl and carotenoid pigments contribute to stability, as discussed in later sections and shown in Figures 3 and 4. Many of the LH1 subunits form direct bonds to the RC. LH1 αβ1 has an extensive interface with PufX, which in turn is strongly bonded to the RC-L subunit (see later section and Figure 7); LH1 αβ2–4 are bonded to the RC-H subunit at H-Ser93 and H-Gly54, and to Trp25 of RC-L on the cytoplasmic side of the complex (Figure 3B), and to L-Trp51 and L-Trp59 on the periplasmic side (Figure 3C). Further round the LH1 ring, Asn42 of LH1 αβ6 bonds to backbone oxygen of RC-H Ala6 (Figure 3D), and LH1 αβ8,9 are hydrogen-bonded to RC-M (Figure 3E). Thus, direct bonding between LH1 and the RC is limited to LH1 αβ2–9, which is also the side of the complex with the shortest distance between LH1 BChls and the RC special pair of BChls (see Figure 4). The arc of subunits 10–14 separates from the RC, opening a gap between the RC and the inner face of LH1 that allows quinones access to the RC Q_B_ site. To ensure that this gap is maintained, protein-Y (Figure 3A, green; also see Figure 6) is inserted between LH1 and the RC subunits at αβ13,14. In general, one side of the LH1 antenna seems to be focused on energy transfer and the other on enabling quinone transport.

**Figure 5.**
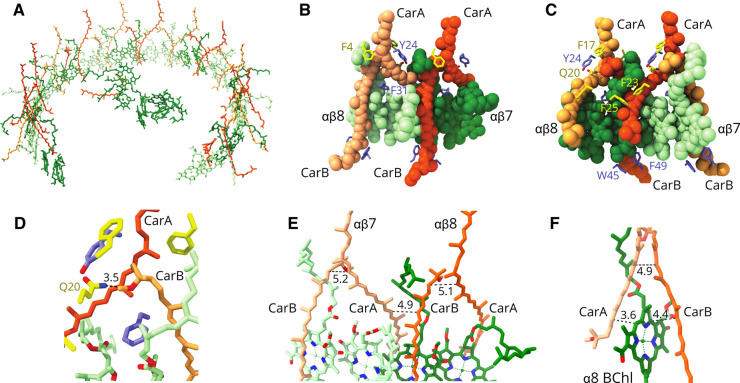
The bacteriochlorophyll and carotenoid pigments in the RC–LH1 monomer. (**A**) The incomplete ring of pigments, with paired BChls belonging to neighbouring LH1 αβ subunits coloured in alternating green/pale green, and paired carotenoids in orange/pale orange. The LH1 pigments enclose the RC pigments, which comprise one carotenoid (orange) and two branches of BChls and bacteriopheophytins, coloured in green. (**B**) Spacefill representation of the pigments bound to two adjacent αβ subunits, at positions 7 and 8 in the rings (see Figure 1D for numbering of LH1 subunits) coloured as in (**A**), showing several interacting aromatic sidechains. (**C**), as in (**B**), but rotated 180°. (**D**) Detailed view of the hydrogen bond from the methoxy of carotenoid B to αGlu20. (**E**) Two carotenoid/BChl pairs from adjacent αβ subunits 7 and 8, showing the close approaches of carotenoids within and between subunits. (**F**) View of an α-bound BChl bridging between carotenoids from adjacent subunits.

**Figure 6.**
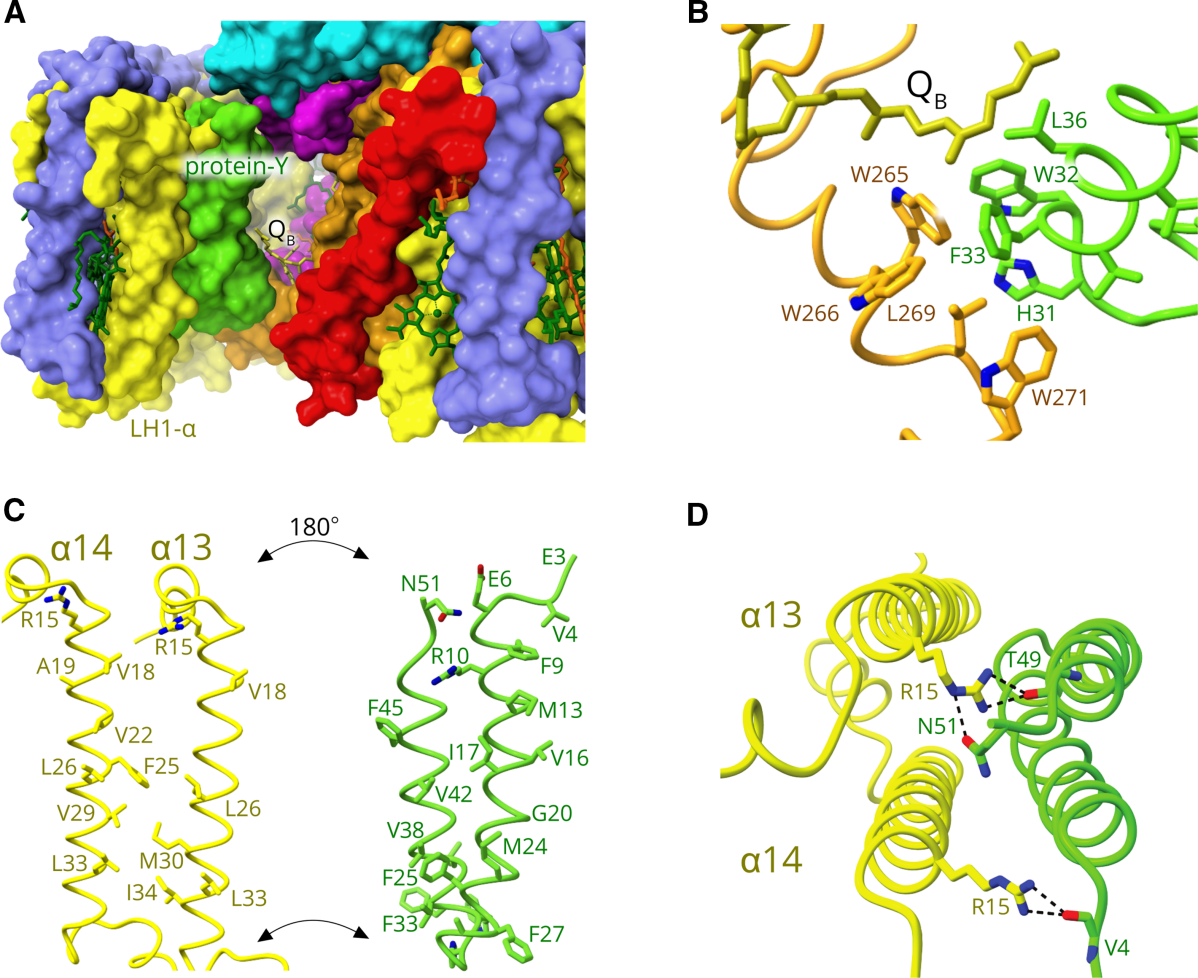
The internal quinone channel created by protein-Y. (**A**) Surface view of the channel, viewed through the gap in the LH1 ring created by PufX (red), which binds to LH1 αβ subunit1. Protein-Y lies against the internal surface of the LH1 complex, at positions 13 and 14. Q_B_ (yellow) is visible in the background. The RC subunits are RC-M (magenta), RC-L (orange) and RC-H (cyan). (**B**) View of the hydrophobic interactions between protein-Y and the RC-L subunit, with the Q_B_ tail also nearby. (**C**) ‘Open book’ format to show the opposing, interacting faces of LH α polypeptides belonging to subunits 13–14 and protein-Y. (**D**) View of the α-protein-Y hydrogen bond interactions on the cytoplasmic side of the complex.

**Figure 7.**
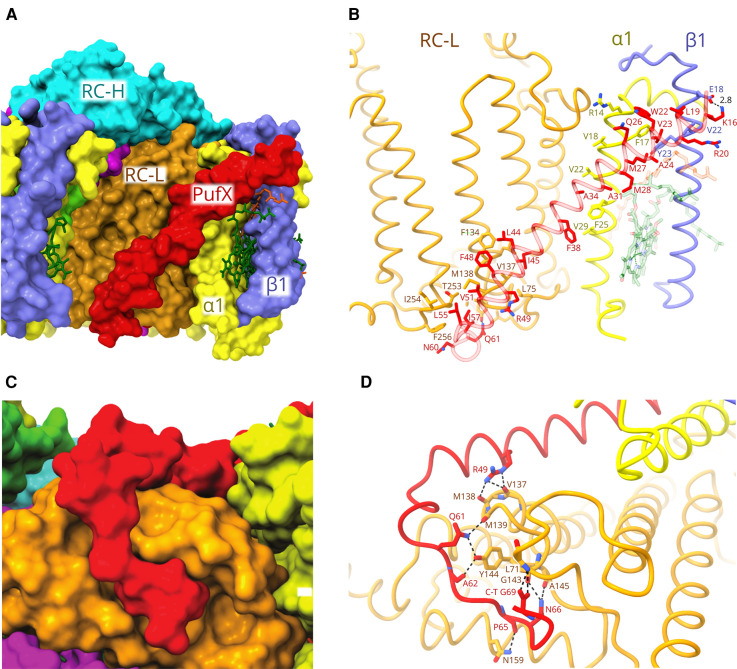
The binding of PufX to RC and LH1 subunits. (**A**) Surface view of PufX (red) viewed through the gap in the LH1 ring. (**B**) The interactions made by the N-terminal half of PufX as it passes across LH1 αβ subunit. The first 14 residues of PufX were not resolved. K16 is hydrogen-bonded to Glu18 of LH1β, but the majority of interactions are van der Waals contacts with LH1α, viewed through the semi-transparent PufX (red). Carotenoids have been removed for clarity. (**C**) The C-terminal part of PufX contacts the RC-L subunit (orange), shown in surface view. (**D**) Details of the interaction in (C), showing the network of hydrogen bonds formed between PufX and RC-L.

### Arrangement of bacteriochlorophylls in the monomeric RC–LH1–PufX complex

There are noticeable differences between LH1 subunits in the RC–LH1 monomer complex. Figure 4 illustrates the open nature of the BChl array, and one consequence of protein-Y pushing LH1 αβ 11–14 away from the RC is a lengthening of the distance between LH1 BChls and the RC special pair. Figure 4A also illustrates the 38.3 Å–50.7 Å range of Mg–Mg distances, which could affect the transfer of excitation energy from LH1 to the RC, and favour energy transfer from BChls attached to LH1 subunits 7–9. The overlapping macrocycles of LH1 BChls (Figure 3A,B) strongly favour ultrafast energy transfer round the ring [24], on timescales much shorter than the 35–50 ps taken for energy to hop from LH1 to the RC [25,26]. Thus, ultrafast energy transfer from, for example, subunit 14 BChls to those on subunit 7, and then hopping 38.3 Å to the RC is, in principle, faster than direct transfer from subunit 14. This amounts to a degree of specialisation within the LH1 ring, and further differences emerge when taking the carotenoids into account (see next section).

Pairs of opposing, excitonically coupled BChls are attached to the transmembrane helices of the LH1 α and β subunits by ligation to α-His32 and β-His39, respectively, and LH1 mutants lacking these ligands do not assemble [27]. The C3 acetyl carbonyl of each BChl is hydrogen-bonded to a Trp in the C-terminal domain of α and β (Figure 4C), and these bonds, to α-Trp43 and β-Trp48, were shown many years ago using a combination of site-directed mutagenesis and Raman spectroscopy [28,29]. This bonding arrangement holds each BChl pair so that their Q_Y_ absorption transitions, which run from ring A to ring C, are approximately parallel to the plane of the membrane. Figure 4C shows that within an LH1 subunit there is some limited overlap between macrocycles at rings C/E, and the A rings of BChls in adjacent subunits overlap almost completely. The intra-subunit and inter-subunit Mg–Mg distances of 9.2 Å and 8.6 Å, respectively, reinforce the impression that BChls in adjacent subunits are positioned for strong excitonic coupling and ultrafast energy transfer round the LH1 assembly. Compared with 850 nm-absorbing LH2 complexes, LH1 BChls are more red-shifted, to ∼875 nm. The coupling between BChls in a subunit shifts the absorption of each pair of BChls from ∼780 nm in solvent to 820 nm, and the LH1 complex can be reversibly dissociated into small, B820 units [30,31]. Experiments that studied the association between αβ(BChl)_2_ B820 complexes of *Rba. sphaeroides*, *in vivo* and *in vitro*, show that the full red shift to 875 nm is achieved when as few as 2 or 3 LH1 αβ subunits associate [32,33].

### Arrangement of carotenoids in the monomeric RC–LH1–PufX complex

The 1:1 ratio of carotenoid:BChl in the *Rba. sphaeroides* LH1 complex was reported over 40 years ago [10] but, despite the two-fold abundance of these pigments relative to other RC–LH1 complexes [4–8], none could be identified in our earlier RC–LH1–PufX structure at 8 Å resolution [12]. Figure 5A shows the arrangement of the spheroidene carotenoids in the LH1 ring, with an interesting heterogeneity in their allocation between LH1 subunits. Each of LH1 subunits 1–13 binds two spheroidene molecules, but the 14th LH1 subunit has no carotenoid. In Figure 5 the two carotenoids assigned to a particular LH1 αβ subunit have a matching shade of orange. One of these paired carotenoids for subunits 1–13 (carotenoid A) corresponds to those resolved in many RC–LH1 structures, and its methoxy group sits next to an α-bound BChl. Then it traverses the membrane running towards the cytoplasmic side of the complex to the N-terminal part of the β-polypeptide belonging to the next subunit along in the ring. Thus, this carotenoid crosses between subunits, but we assign the carotenoid according to its proximity to the β-polypeptide N-terminus. The second carotenoid (carotenoid B) in subunits 1–13 has not been found in other LH1 complexes, and it is displaced in the plane of the membrane, with an inverted orientation relative to carotenoid A (Figure 5B,C). The binding of this second carotenoid is made possible by displacement of the α-BChl phytol tail. The spacefill representations in Figure 5B,C emphasise the close packing of LH1 pigments, with carotenoid–carotenoid, carotenoid–BChl and BChl–BChl contacts evident. A hydrogen bond is tentatively assigned between the methoxy group of carotenoid B and α-Gln20 (Figure 5D) towards the cytoplasmic side of the complex. Carotenoid B is in van der Waals contact with carotenoid A (Figure 5E,F); C3 of carotenoid B is 5.1–5.2 Å from C20 of carotenoid A, and C7 of carotenoid B is 4.9 Å from C11 of carotenoid B. Given that spheroidene has 10 conjugated C = C bonds, from C3–C22, the LH1 structure shows that the tight packing of carotenoids brings the π-conjugated C = C bond systems of carotenoids A and B into contact, not only within a subunit but also between them (Figure 5E). The coupling between carotenoids A and B is reinforced by an intervening α-bound BChl, which provides a π-conjugated bridging system (Figure 5F).

### Protein-Y and the internal quinone channel

The presence of the protein-Y polypeptide was unexpected, yet Figure 6 shows clearly that it has an important function. Figure 6A shows that PufX lies diagonally across the gap in the LH1 ring, preventing its closure, but still leaving the possibility that the loose, open end of the LH1 assembly at position 14 could curve in towards the RC and impede quinone traffic. This is possible because the linkages between LH1 αβ subunits are flexible, as shown by atomic force microscopy (AFM) studies of LH1 in membranes from a mutant with no RCs [34,35]. LH1-only assemblies can form ellipses, spirals and rings of variable size, so the linkage between LH1 αβ 13 and 14 is likely to allow some movement unless a protein, protein-Y in this case, holds the LH1 gap open. The view through the gap in LH1 towards protein-Y shows that it lies against the inside surface of the α polypeptides belonging to subunits 13 and 14 (Figure 6A). Part of the tail of Q_B_ is visible in the background because protein-Y maintains an internal opening by making contacts with W265, W266, L269 and W271 on the RC-L subunit (Figure 6B). These contacts are near the loop region towards the middle section of protein-Y, which joins two transmembrane helices (Figure 6C, green) creating a hydrophobic hairpin structure. Supplementary Figure S5 shows that there is another structurally defined quinone in the vicinity of RC Q_B_, Q_2_, which could be a quinone about to enter or leave the RC Q_B_ site. Q_2_ has no protein binding site, as with other such ‘free’ quinones identified in RC–LH1 structures in *Rhodopseudomonas (Rps.) palustris*, *Thermochromatium (Tch.) tepidum, Rhodospirillum (Rsp.) rubrum* and *Rba*. *veldkampii* [4–8]. Supplementary Figure S5 also shows that the region between the RC and the inside face of LH1 subunits 3–7 is occupied by the lipids cardiolipin and phosphatidyl ethanolamine, the packing of which could help to confine the diffusion of quinones to the space between the RC and LH1 subunits 11–14.

The transmembrane regions of LH1 α13 and α14 are shown with protein-Y in an ‘open book’ format that shows the hydrophobic sidechains that form the α13–α14–protein-Y interface (Figure 6C). The view perpendicular to this interface from the cytoplasmic side (Figure 6D) shows that Val4, Thr49 and Asn51 of protein-Y form hydrogen bonds with N-terminal LH1 α-Arg15 residues. Thus, protein-Y is anchored in place at the N-terminus of LH1 α, then by the hydrophobic α1–α2–protein-Y interface, and finally to the RC-L subunit, forming a stable channel on the RC side of protein-Y for quinones to enter and leave the RC Q_B_ site (Figure 6A). This channel created by the insertion of protein-Y widens the arc of LH1 subunits at positions 11–14; comparison with a RC–LH1_15_–PufX complex from *Rba. veldkampii* with no protein-Y (Supplementary Figure S6A) shows how the absence of a protein-Y allows LH1 to curl in towards the RC. This inward movement is even more pronounced in Supplementary Figure S6B, which compares the RC–LH1_14_–PufX–protein-Y complex of *Rba. sphaeroides* with the RC–LH1_14_–protein-W complex of *Rps. palustris*, and in each case the LH1 subunits of *Rba. veldkampii* and *Rps. palustris* encroach on the position occupied by protein-Y in *Rba. sphaeroides*. This point will be discussed later, but this comparison supports the idea that protein-Y is a modification that establishes a stable quinone channel.

### PufX and the external quinone channel

PufX forms a barrier to the complete encirclement of the RC by LH1 subunits. Figure 7A,B shows that PufX lies diagonally across the complex, traversing the membrane and making an extensive series of interactions with three different polypeptides. The N-terminal part of PufX is held to the first LH1 subunit on the cytoplasmic side of the complex by a hydrogen bond to Glu18 of LH1-β1. The density map did not allow assignment of the first 14 residues of PufX, so we cannot discount further interactions of the PufX N-terminus with LH1. As PufX crosses the first LH1β polypeptide, then LH1 α, there is a PufX-αβ interface that consists of several close hydrophobic contacts (Figure 7B), and the C-terminal part of PufX wraps round RC-L on the periplasmic side (Figure 7C), held in place by a network of hydrogen bonds (Figure 7D). Thus, once anchored in position by attaching to RC-L, we suggest that PufX is the point where LH1 assembly begins, when the first LH1 αβ BChl_2_carotenoid_2_ unit attaches to the N-terminal half of PufX, as in Figure 7B.

## Discussion

AFM of membranes from photosynthetically grown cells of *Rba. sphaeroides* shows that the RC–LH1 core complexes are mainly dimeric [36], and computational models of chromatophore vesicles reflect the low proportion of monomers [1–3,37]. Mild treatment of such membranes with β-DDM can fractionate membranes and reveal populations of monomers and dimers [38], and this type of analysis shows that the type of carotenoid in the complex appears to influence the proportion of monomers observed. In oxygen-limited conditions RC–LH1 monomers represent more than half of the core complexes in the membrane, whereas dimers form the majority of the cores in anaerobic, phototrophically grown cells [39,40]. Thus, the level of oxygen appears to influence the balance between monomers and dimers, which is likely linked to the oxygen-dependent switch from spheroidene to spheroidenone, although we do not know how this affects the structure of the complex, nor do we understand its functional significance.

To obtain a homogeneous population of monomers for biochemical and structural analysis we used a mutant of *Rba. sphaeroides* in which the C-terminal Arg53 residue of PufX was altered to Leu [9]. This single change has no effect on the rate of photosynthetic growth [9], and the structure shows that Arg53 is in a part of PufX that is not involved in extensive interactions with LH1 or RC subunits, nor are there any interactions of Arg53 with functionally essential cofactors such as carotenoids, BChls or quinones. Thus, the effect of the Arg53 to Leu mutation is confined to altering the proportion of monomers and the structure obtained here is directly relevant to the monomers found in wild-type membranes. The present monomer structure at 2.5 Å resolution shows the details of interactions between polypeptides, BChls and carotenoids necessary for light harvesting and photochemistry, as well as revealing the contributions made by protein-Y and PufX to the efficient passage of quinones across the LH1 ring. However, the way in which PufX mediates the association of monomers to form a dimeric complex [9,12,41,42] is not known and this information requires a higher resolution structure of the dimeric RC–LH1 complex.

### The assembly and function of the LH1 complex

The structure of the monomeric RC–LH1–PufX–protein-Y complex shows an interesting variation in roles for the 14 LH1 αβ subunits, as opposed to the relatively undifferentiated LH1 subunits found in the RC–LH1_16_ complex of *Rsp. rubrum*, for example [6,7]. In *Rba. sphaeroides*, it has been shown that PufX is incorporated into developing core complexes relatively early in the assembly sequence, followed by LH1 [43]. We suggest that this process is initiated when the C-terminus of PufX forms the bonding network with the RC-L subunit shown in Figure 7B,D. PufX lies diagonally across the transmembrane section of RC-L making many van der Waals contacts (Figure 7B), leaving the N-terminal part of PufX free to bind the first LH1 αβ BChl_2_carotenoid_2_ subunit (Figure 7B). Four different types of interaction guide the incorporation of incoming LH1 αβ subunits within the growing complex: a given LH1 αβ subunit can bind by interacting directly with a preexisting one, as shown in Figure 2, and particularly for the first nine LH1 αβ subunits there are also multiple associations with the RC (Figure 3). Rings A of BChls in adjacent subunits overlap (Figure 4), and carotenoid A crosses between subunits to provide another stabilising interface (Figure 5). Of these interactions, those involving the RC do not appear to be critical for association of LH1 subunits because *in vitro* reconstitution experiments show that LH1 αβBChl_2_ (B820) subunits can oligomerise to form larger, red-shifted assemblies [30]. However, the RC has an important template role and an AFM study showed that in its absence LH1-only mutants adopt a range of sizes and shapes [34,35]. As the incremental assembly of an LH1 antenna round the RC continues the BChls on subunit 7 make the closest approach, 38.3 Å, to the special pair BChl in the RC. Thus, LH1 αβ7 could represent a preferential site for receiving excitation energy from other points in the LH1 ring further away from the RC (Figure 4A) [44], given that energy transfer between excitonically coupled BChls in the LH1 array is extremely rapid, on a timescale of 50–150 fs [24]. Minimising the LH1–RC distance could be advantageous, given that this is the rate limiting step in energy trapping [44] due to the inverse sixth power dependence of Förster resonance energy transfer [45].

Further round the LH1 ring, subunit 9 is the last one that binds to the RC and as the arc of LH1 extends further round towards the RC Q_B_ site subunits 11—14 begin to separate from the RC, creating a space between the complexes that allows movement of quinones. By the time that subunit 14 has been added, it appears that there is some instability in the assembly, and this subunit is the only one that binds no carotenoids, and lacks a partner, either PufX or another LH1 subunit. It is possible that carotenoids at LH1 αβ14 are only weakly bound and are lost during purification of the complex or are sufficiently flexible to be unresolveable in the map. The flexible contacts between LH1 subunits are evident from the multiple conformations of LH1 arrays imaged by AFM [34,35], and it could be possible for subunits 11–14 to bend inwards, impairing quinone diffusion, were it not for the presence of protein-Y. The timing of incorporation of this hitherto undescribed protein into the RC–LH1–PufX complex is unknown.

### Balancing the absorption of light and quinone traffic

**Figure 8.**
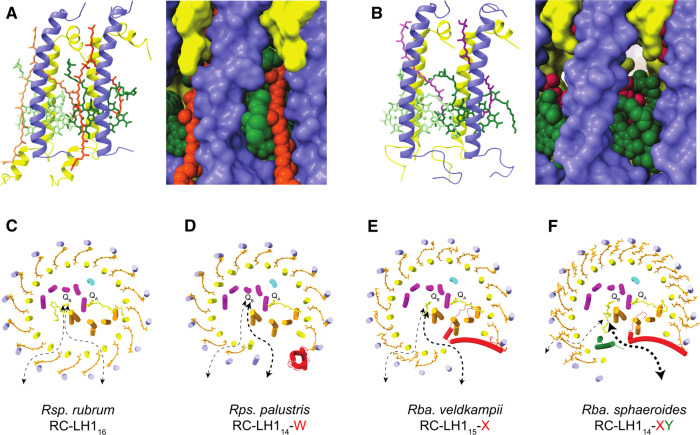
Pores and channels for quinone traffic in RC–LH1 complexes. (**A** and **B**) Comparison of *Rba. sphaeroides* RC–LH1–PufX–protein-Y (**A**) and *Rps. palustris* RC–LH1–protein-W (**B**) complexes, viewed in the plane of the membrane from the exterior of the complex, as ribbons (left) and in spacefill representation (right) for each complex. (A) LH1 αβ7–αβ8 pairs (LH1α in yellow and LH1β in blue) from the *Rba. sphaeroides* RC–LH1–PufX–protein-Y complex, with BChls in green and four carotenoids (spheroidene) in orange. (**B**) As in (**A**), but from the *Rps. palustris* RC–LH1–protein-W complex, and the two carotenoids are spirilloxanthin. (**C**–**F**) Projection views of four RC–LH1 structures, showing the possible pores and channels for quinone traffic in complexes from (**C**) *Rsp. rubrum*, (**D**) *Rps. palustris,* (**E**) *Rba. veldkampii and* (**F**) *Rba. sphaeroides.* Colours: RC-M (magenta), RC-L (orange), RC-H (cyan), protein-Y (green), PufX/protein-W (red). The thickness of the arrows suggests the relative extents of quinone diffusion through PufX/protein-W-mediated gaps in the LH1 ring and via pores between subunits.

Figure 8A,B shows a comparison of LH1 subunits viewed from the exterior of the complex in the plane of the membrane. The spacefill representation shows that there are no gaps within or between *Rba. sphaeroides* subunits large enough for quinone/quinol passage (Figure 8A) but a large pore is clearly seen for *Rps. palustris* LH1, and indeed for other such complexes where there is only one carotenoid per LH1 αβ subunit (Figure 8B). Therefore, to create the additional carotenoid binding site, it appears that the quinone pore common to single carotenoid binding LH1s has been repurposed to accommodate a second carotenoid in *Rba. sphaeroides*. However, despite the binding of the second carotenoid and the absence of an obvious pore in Figure 8B, there must still be transient openings in the LH1 ring for quinone traffic, because a study of the kinetics of quinone turnover showed that there is only a two-fold penalty for closing the LH1 ring, relative to a complex with PufX [46]. Figure 8C–F show projection views that compare the *Rsp. rubrum* RC–LH1, *Rps. palustris* RC–LH1–protein-W, *Rba. veldkampii* RC–LH1–PufX, and *Rba. sphaeroides* RC–LH1–PufX–protein-Y complexes. These figures illustrate four structural options that have evolved for allowing quinones to diffuse in and out of the LH1 ring, enabling turnover of the cytochrome *bc*_1_ complex [1].

In the minimal RC–LH1 complex, seen in structures from *Rsp. rubrum*, *T. tepidum*, and in the RC–LH1 complex *Rps. palustris* lacking protein-W [4–8], LH1 completely surrounds the RC and quinones must diffuse through small pores in the LH1 ring, such as the one in Figure 8B, by means of ‘breathing motions’ [47,48]. In fact, even among an apparently undifferentiated set of pores such as those in the *Rsp. rubrum* LH1 complex (Figure 8C) there is a preferred location in the LH1_16_ ring for quinone transport, designated as a Q_P_ site (not shown) [6], which is also found in the *Blastochloris viridis* RC–LH1 complex [49]. Here, the head group of the Q_P_ quinone forms presumably temporary bonds with the RC-L subunit and the LH1α polypeptides, and we have suggested that the Q_P_ binding pocket prepares a transiently docked quinone for passage through an adjacent pore between LH1 subunits [49].

Figure 8D–F shows that in some bacteria the LH1 ring is interrupted by a protein such as protein-W or PufX that prevents LH1 ring closure, creating a gap for quinone traffic [5,8,12,23,50]. In these cases, as in the *Rps. palustris* (Figure 8D) and *Rba. veldkampii* (Figure 8E) complexes, inter-subunit LH1 pores have been retained, whereas in *Rba. sphaeroides* (Figure 8F) the pores are occluded by the extra carotenoid in each LH1 subunit, seen in Figure 8A. Despite this apparent pore closure the rate of quinol diffusion to the cytochrome *bc*_1_ complex is slowed only two-fold in a PufX-minus mutant [46], so transient gaps can still allow some quinone traffic across the LH1 subunits. Nevertheless, the single wide opening created by PufX presents the only efficient route for transporting quinones across the LH1 ring and as a result PufX-minus mutants of *Rba. sphaeroides* cannot photosynthesise [51–53], unless the LH1 ring is also absent [54]. The 13 extra carotenoids that close pores in the *Rba. sphaeroides* RC–LH1 complex, relative to the *Rba. veldkampii* complex, have the advantage of providing a higher level of photoprotection [55]. Also, increased absorption by carotenoids in the 500–600 nm region of the spectrum provides more energy to drive faster turnover at the RC, accelerating quinone diffusion in and out of the complex. Protein-Y appears to be a response to the need for improved quinone diffusion; it binds to transmembrane regions on the inside face of the LH1 complex at α13 and α14, and it also forms hydrogen bonds to N-terminal residues of these LH1α polypeptides. Further binding to the RC-L subunit anchors protein-Y in place, creating a stable internal channel for quinones between the inner face of protein-Y and RC-L (Figure 4A). In its absence, the arc of LH1 subunits 11–14 could curve towards the RC, and potentially restrict quinone diffusion by narrowing the external opening (Supplementary Figure S5). The combination of binding more LH1 carotenoids and acquiring protein-Y could be advantageous for *Rba. sphaeroides* in high light conditions, which would benefit from enhanced photoprotection and the capacity to absorb and use more solar energy. However, light-driven energy metabolism is a series of interlinked reactions and increasing the capacity or rate of one process imposes more demands on another downstream; the PufX–protein-Y combination in *Rba. sphaeroides* avoids the possibility of restricted quinone diffusion across the LH1 barrier, and the limiting step for cyclic electron flow becomes the rate of turnover at the nearby cytochrome *bc*_1_ complex [1,3].

## Data Availability

The cryo-EM density map has been deposited in the World Wide Protein Data Bank (wwPDB) under accession code EMD-13441 and the coordinates have been deposited in the Protein Data Bank (PDB) under accession number 7PIL. The mass spectrometry proteomics data have been deposited to the ProteomeXchange Consortium via the PRIDE partner repository (http://proteomecentral.proteomexchange.org) with the data set identifier PXD028048.

## Open Access

Open access for this article was enabled by the participation of University of Sheffield in an all-inclusive *Read & Publish* pilot with Portland Press and the Biochemical Society under a transformative agreement with JISC.

## Abbreviations

AFM: atomic force microscopy
BChl: bacteriochlorophyll *a*
cryo-EM: cryogenic electron microscopy
*Rba.*: *Rhodobacter*
RC–LH1: reaction centre light-harvesting complex 1
*Rps.*: *Rhodopseudomonas*
*Rsp*.: *Rhodospirillum*
*Tch.*: *Thermochromatium*
