# Supplementary material for: Cryo-EM structure of the monomeric Rhodobacter sphaeroides RC–LH1 core complex at 2.5 Å
Source: Biochem J. 2021 Oct 21;478(20):3775–90. doi: 10.1042/BCJ20210631 (PMC8589327; doi:10.1042/BCJ20210631)
Supplement: Supplementary Figures S1-S6 and Table S1 [file BCJ-478-3775-s1.pdf]

Supplementary material

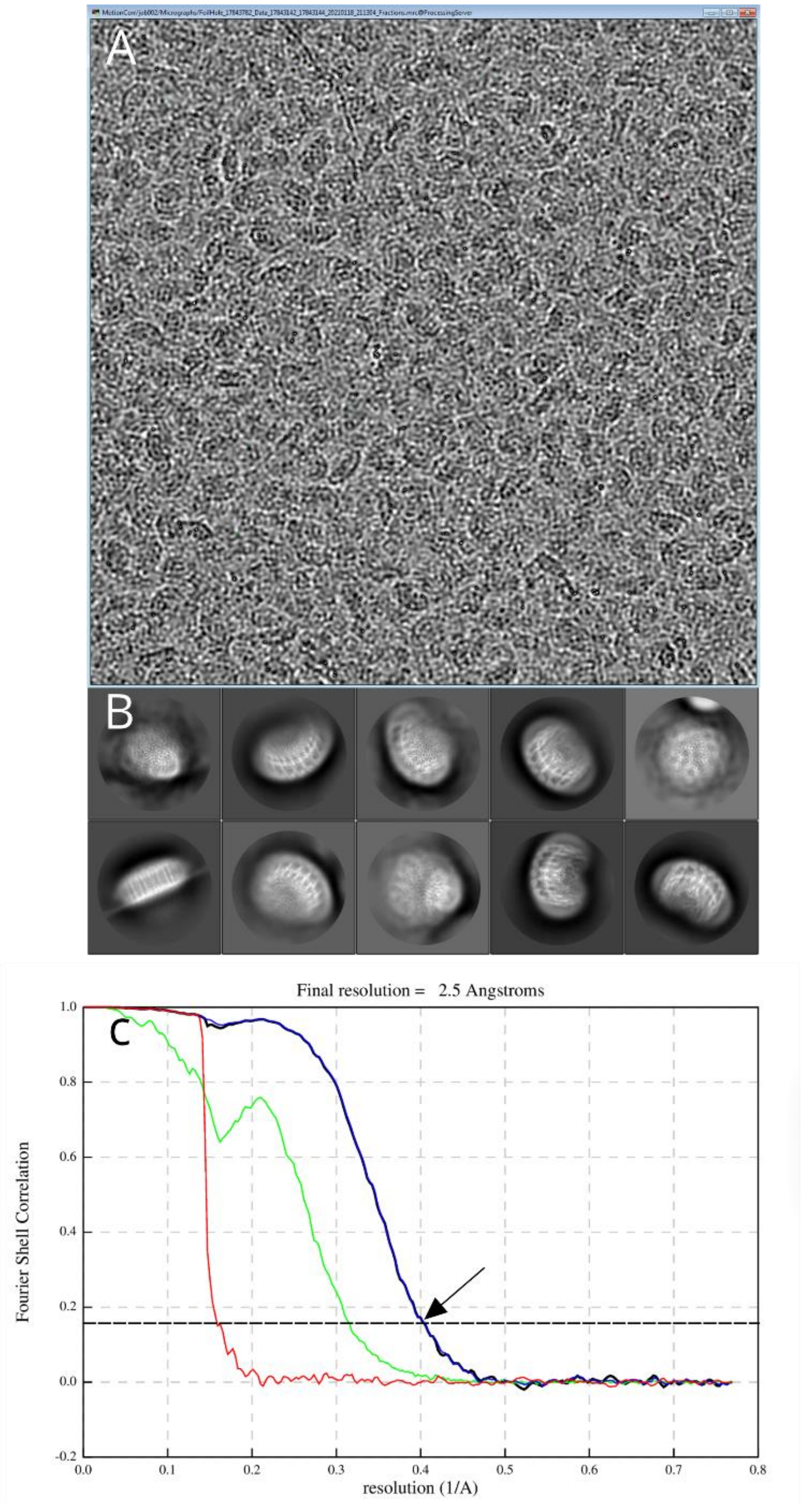

Figure S1. Cryo-EM image of the RC-LH1-PufXY complex from *Rba. sphaeroides* and resolution calculation of 3D map of the complex. (A) A selected cryo-EM micrograph after motion correction. The pixel size of the image is 4k x 4k, corresponding to 266 x 266 nm at the specimen level. A sigma contrast of 3 was applied. (B) Selected 2D classes, box size 25 nm, showing different views of the complex. (C) Fourier shell correlations (black, corrected; green, unmasked; blue, masked; red, phase randomised). The dashed line shows 0.143 FSC, with an arrow pointing to the global resolution of the map at 2.5 Å.

MPEVSEFAFRLMMAAVIFVGVGIMFAFAGGHWFVGLVVGGLVAAFFAATPNSN

Figure S2. Identification of Rsp_7571 (proposed name: protein Y) in isolated RC-LH1 complexes by mass spectrometry. Proteolytic peptides generated by pepsin and identified by database searching are indicated by red lines. Amino acids in blue are fitted into the cryo-EM map.

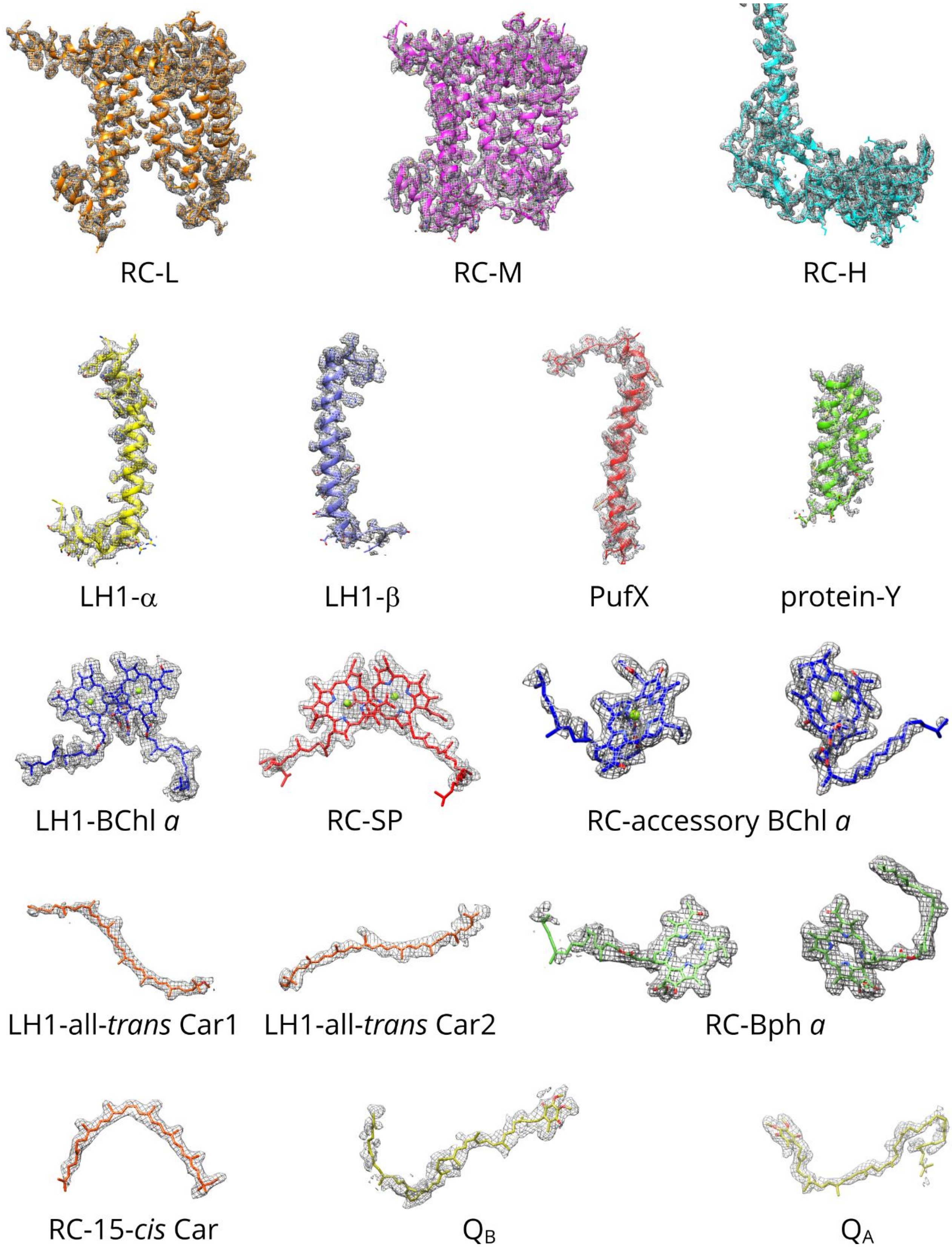

Figure S3. Cryo-EM densities and structural models of polypeptides and pigments in the monomeric RC-LH1 complex from *Rba. sphaeroides*. Atomic models of components of the complexes are fitted into their respective density maps, taken from the final refined model. RC-SP are the reaction centre special pair of BChls.

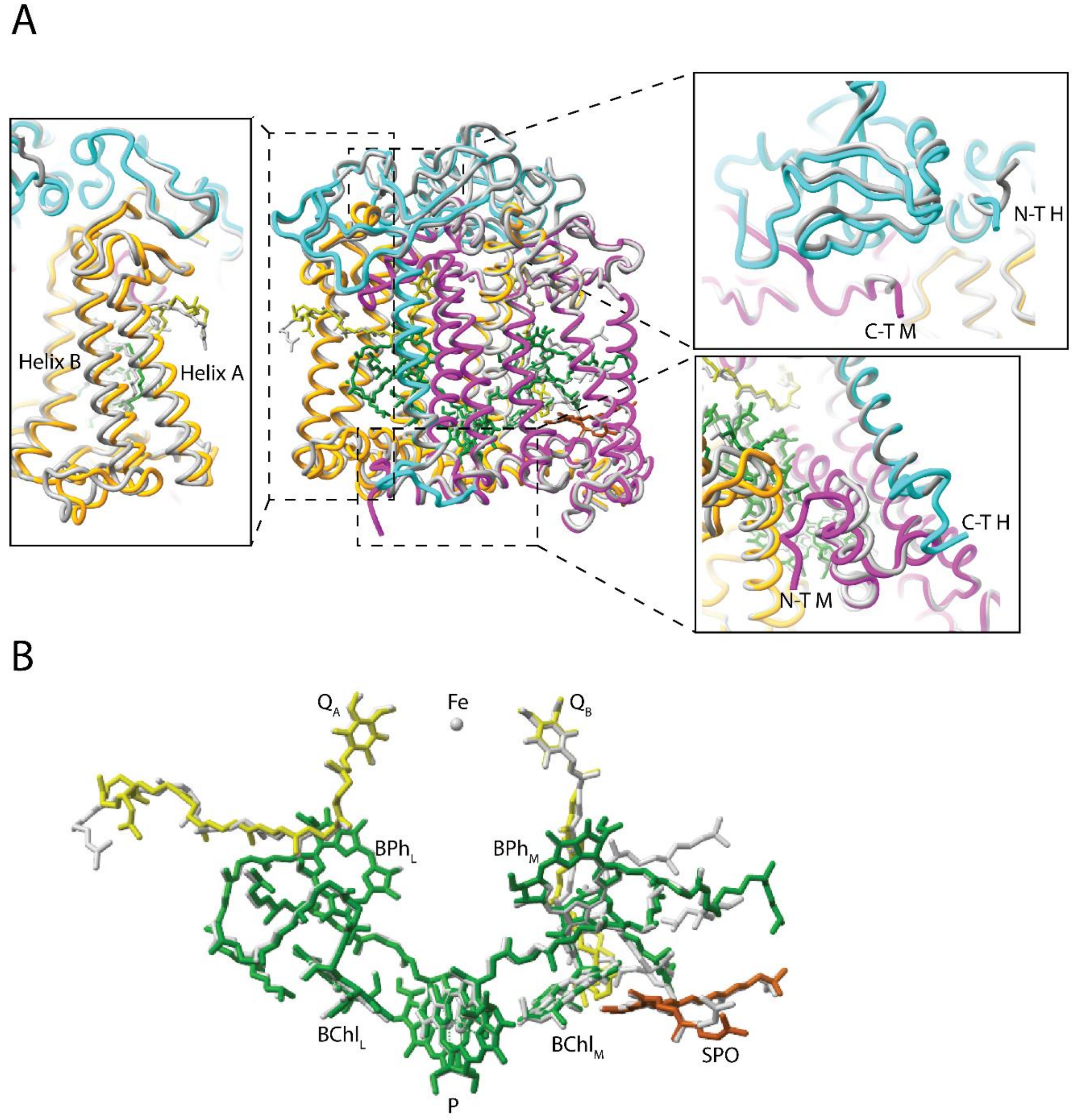

Figure S4. Comparison between RC structures obtained from cryo-EM of the RC-LH1-PufXY complex. (A) Overlaid structures from cryo-EM (RC-L magenta; RC-M, orange; RC-H, cyan), and a structure (3I4D) obtained using X-ray crystallography (grey). Three regions are highlighted in boxes to show the small deviations between the structures. N-T and C-T indicate N- and C-termini, respectively. (B) Overlaid pigments and cofactors, with those from 3I4D in grey.

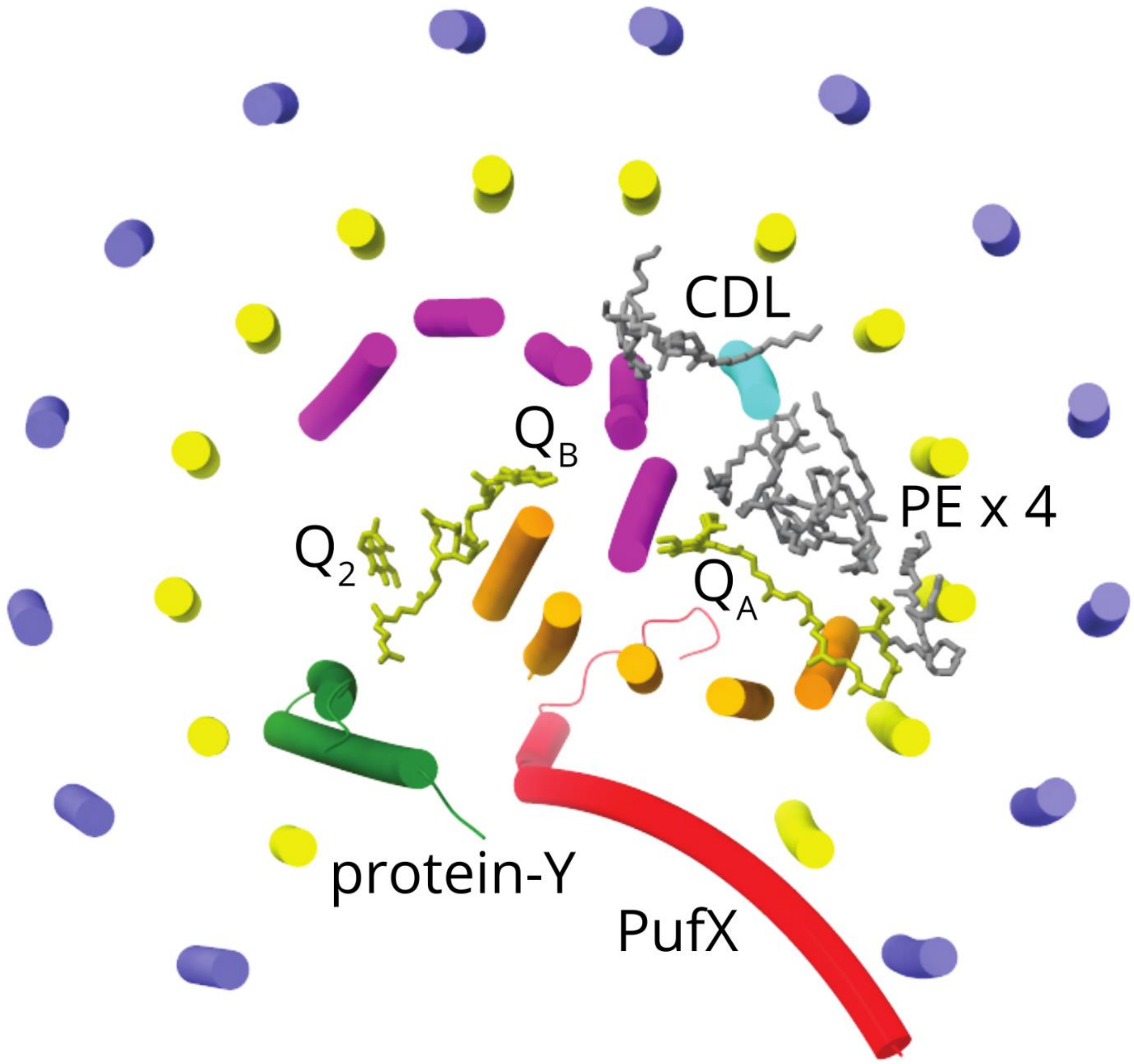

Fig. S5. Projection view of the RC-LH1 monomer complex of *Rba. sphaeroides* in a simplified form that omits LH1 and RC pigments, and which represents alpha helices as coloured tubes. The colours are LH1 α (yellow), LH1β (blue) RC-L orange), RC-M (magenta), quinones (dark yellow) and lipids (grey). CDL is cardiolipin and PE is phosphatidyl ethanolamine. Q_A and Q_B are quinones bound to sites in the RC and Q_2 is a quinone with no protein binding site.

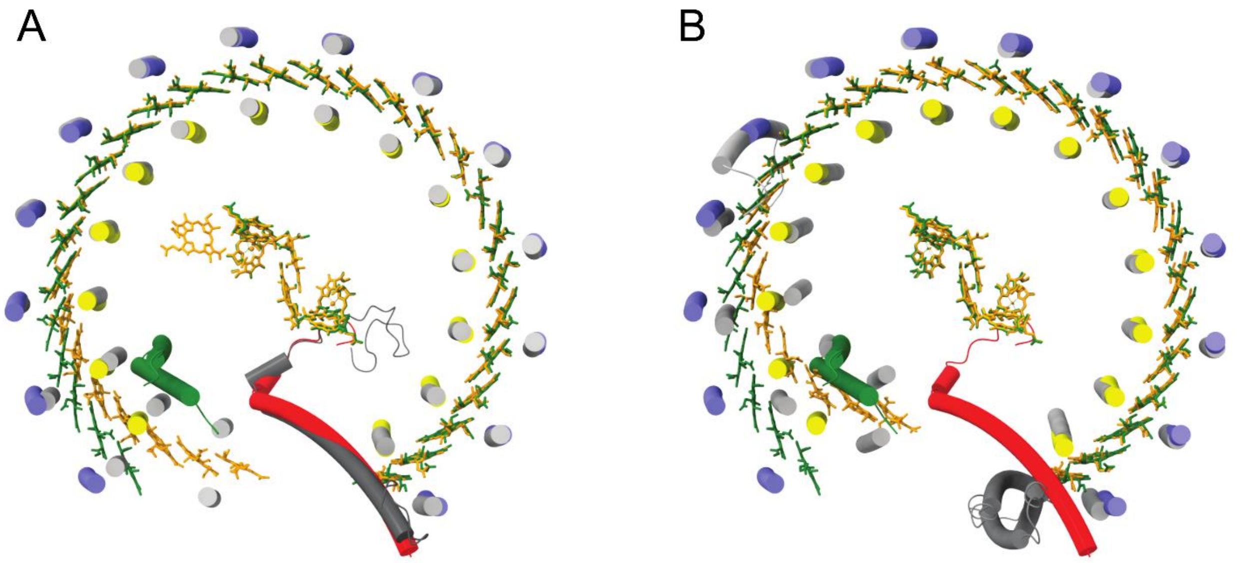

Rba. sphaeroides RC-LH1$_{14}$-XY (coloured) *vs* *Rba. veldkampii* RC-LH1$_{15}$-PufX (grey).

Rba. sphaeroides RC-LH1$_{14}$-XY (coloured) *vs* *Rps. palustris* RC-LH1$_{14}$-W (grey).

Figure S6. Comparison between the LH1 rings of *Rba. sphaeroides*, *Rba. veldkampii* and *Rps. palustris*. In each case, the coloured structure and green BChls are from *Rba. sphaeroides,* and the other complex is in grey, with orange BChls and RC pigments. (A) The two PufX structures align well, and the *Rba. veldkampii* BChls form a more extensive ring. Protein Y is in green. (B) Comparison with the core complex variant of *Rps. palustris* with 15 αβ subunits and protein-W (grey).

Table S1: Cryo-EM data acquisition, model refinement and validation statistics.

Protein source	Photosynthetic bacterium
Data collection and processing	
Microscope	ThermoFisher Titan Krios G3i
Voltage (kV)	300
Camera	Falcon 4
Energy filter	No
Energy filter slit width	No
Magnification	120,000
Defocus range (µm)	-0.8 to -2.2
Mean defocus (µm)	-1.8
Pixel size (Å)	0.65
Electron flux (e^-/ Å^2/s)	3.71
Electron fluence (e^-/ Å^2)	44.99
Exposure time (sec/frame)	0.29
Electron fluence per frame (e/ Å^2/frame)	1.07
Number of frames per movie	42
Number of movies used	4859
Initial no. particle images	1,057624
Final no. particle images	250,613
Symmetry imposed	C1
Local resolution range	2.4 to 2.6
Resolution of unmasked reconstruction (Å, FSC=0.143)	3.1
Resolution of masked reconstruction (Å, FSC=0.143)	2.5
Specimen temperature	~80K
Particle box size	(512 px)^2
Refinement and validation	
Refinement package	COOT, ISOLDE, PHENIX
Initial model	PDB 4V9G
Model resolution (Å, FSC=0.5)	2.5
Map sharpening B factor (Å^2)	-55.07
Model composition	
Non-hydrogen atoms	22483
Protein residues	2291
Molecular weight (kD)	315.8
Protein B factor (Å^2)	23.6
RMS deviations	
Bond length (Å)	0.004
Bond angle (^{0})	0.893
Validation	
MolProbity score	9.5
Clashscore	1.9
Rotamer outliers (%)	0.21
EMRinger score	5.68
Cb deviations (%)	0.00
CaBLAM outliers (%)	0.6
Ramachandran plot	
Favoured (%)	98.34
Allowed (%)	1.66
Disallowed (%)	0.00
Ramachandran Z-score	1.06
PDB ID	7PIL
EMDB ID	EMD-13441
